# Schmallenberg virus challenge models in cattle: infectious serum or culture-grown virus?

**DOI:** 10.1186/1297-9716-43-84

**Published:** 2012-12-11

**Authors:** Kerstin Wernike, Michael Eschbaumer, Angele Breithaupt, Bernd Hoffmann, Martin Beer

**Affiliations:** 1 Institute of Diagnostic Virology, Friedrich-Loeffler-Institut (FLI), Suedufer 10, Greifswald, Insel Riems, 17493, Germany; 2 Department of Experimental Animal Facilities and Biorisk Management, Friedrich-Loeffler-Institut (FLI), Suedufer 10, Greifswald, Insel Riems, 17493, Germany; 3 Faculty of Veterinary Medicine, University of Calgary, Calgary, Canada; 4 Present address: Department of Veterinary Pathology, Faculty of Veterinary Medicine, Freie Universität Berlin, Berlin, Germany

## Abstract

Schmallenberg virus (SBV), discovered in Europe in 2011, causes mild transient disease in adult ruminants, but fetal infection can lead to severe malformation in cattle, sheep and goats.

To elucidate the pathogenesis of this novel orthobunyavirus, considerable efforts are required. A reliable and standardized infection model is essential for in vivo studies. In the present study, two groups of four cattle were inoculated with either serum passaged in cattle only or cell culture-grown virus. The replication of culture-grown SBV in cattle was reduced compared to virus inoculated via infectious serum. In a second experiment, the infectious serum was titrated in calves; the tested batch contained 10^2.83^ infectious doses per mL. Hence, serum-borne virus that was only passaged in the natural host is a suitable option for a standardized SBV infection model.

## Introduction

In 2011, Schmallenberg virus (SBV), an orthobunyavirus related to viruses of the Simbu serogroup, was discovered in Germany [1,2]. In adult cattle, SBV infection is associated with mild transient disease, but it can lead to severe fetal malformations when pregnant cows and ewes are exposed in early to mid pregnancy [3]. Currently, the knowledge about SBV is very limited; a reliable and highly standardized infection model is required to elucidate its pathogenesis and to test potential vaccines.

To this end, the present study compares the induction and progression of viremia in cattle for the two different inocula, (i) an SBV field strain that was only passaged in cattle (delivered as infectious serum) as well as (ii) virus grown in tissue culture for a maximum of 4 passages. In addition, the infectious serum was titrated in calves to quantify its infectivity.

## Materials and methods

All experimental protocols were reviewed by a state ethics commission and have been approved by the competent authority (State Office for Agriculture, Food Safety and Fisheries of Mecklenburg-Vorpommern, Rostock, Germany, ref. LALLF M-V TSD/7221.3-1.1-004/12). All animals were between 9 and 18 month of age.

### Infectious serum as inoculum

In a previous study, a single calf had been subcutaneously inoculated with blood samples from four diseased cattle [1] of the same outbreak series that were positive in an SBV real-time quantitative reverse transcription PCR (RT-qPCR). EDTA-treated whole blood, taken from this calf 4 days after infection (dpi) was subcutaneously injected into two further calves (C01, C02). Both inoculated animals were PCR-positive for the first time at 2 dpi, and one developed diarrhea for several days. Half a liter of serum from each calf was collected at 3 dpi, divided into portions of 1 mL and stored at −70°C until it was used in the subsequent experiments.

The sera contained 2.0 × 10^3^ and 1.3 × 10^3^ 50% tissue culture infectious doses per mL (TCID_50_/mL), respectively, as determined by end-point titration on baby hamster kidney (BHK) cells (cell line L0164, Collection of Cell Lines in Veterinary Medicine, Friedrich-Loeffler-Institut, Insel Riems, Germany). Both sera were tested free of any bacterial contamination. Using sensitive real-time PCR protocols, it could be shown that they did not contain detectable nucleic acids of bovine herpesvirus type 1 [4], pestiviruses [5], Rift Valley fever virus [6], bluetongue virus, foot-and-mouth disease virus or epizootic hemorrhagic disease virus (unpublished assays).

### Cell culture supernatant as inoculum

SBV was isolated from the blood of an infected cow as previously described [1]. After initial isolation on KC cells (cell line L1062; derived from Culicoides variipennis midges [7]) the virus was passaged in BHK cells, then in KC and again in BHK cells. The infectivity was determined by end-point titration on BHK cells.

### Animal experiments

#### Comparison between infectious serum and cell culture supernatant

Twelve SBV-naive calves were assigned to three groups of four animals each. Calves in group 1 (C03-C06) were subcutaneously injected with 1 mL of serum obtained from C01, while calves in group 2 (C07-C10) received 2 × 10^7^ TCID_50_ of culture-grown virus. Animals in group 3 (C11-C14) were injected with phosphate-buffered saline (PBS) and kept as controls.

Following the inoculation, the animals were monitored for the presence of clinical signs every day, rectal body temperatures were measured daily and serum samples were taken daily during the first eight days and weekly thereafter. RNA was extracted using the MagNA Pure LC Total Nucleic Acid Isolation Kit for automated extraction (Roche Diagnostics Deutschland GmbH, Mannheim, Germany) according to the manufacturer’s recommendations. SBV genome load in the samples was determined by S segment-specific RT-qPCR as described previously [8] with an external standard based on the S genome segment; at its peak, the difference between the groups was statistically evaluated with a *t*-test. Serological data was collected at weekly intervals using a commercially available ELISA kit (ID Screen® Schmallenberg virus Indirect ELISA kit, IDvet, Montpellier, France). A diverse panel of tissue samples (e.g. lymphnodes, spleen) was taken at necropsy on days 24 or 25; they were homogenized and tested for the presence of SBV RNA by RT-qPCR.

#### In vivo titration of infectious serum

In a subsequent in vivo titration experiment two calves received the undiluted serum of C01 (group A, C15-C16), while groups of three calves each were given dilutions of 1/10 (group B, C17-C19), 1/100 (group C, C20-C22) and 1/1000 (group D, C23-C25) in sterile PBS, respectively. Serum samples were taken at days 1 to 7 and 21; they were tested for SBV RNA and SBV-specific antibodies as described above.

## Results

### Comparison of cell culture supernatant and infectious serum as inoculum

**Figure 1:**
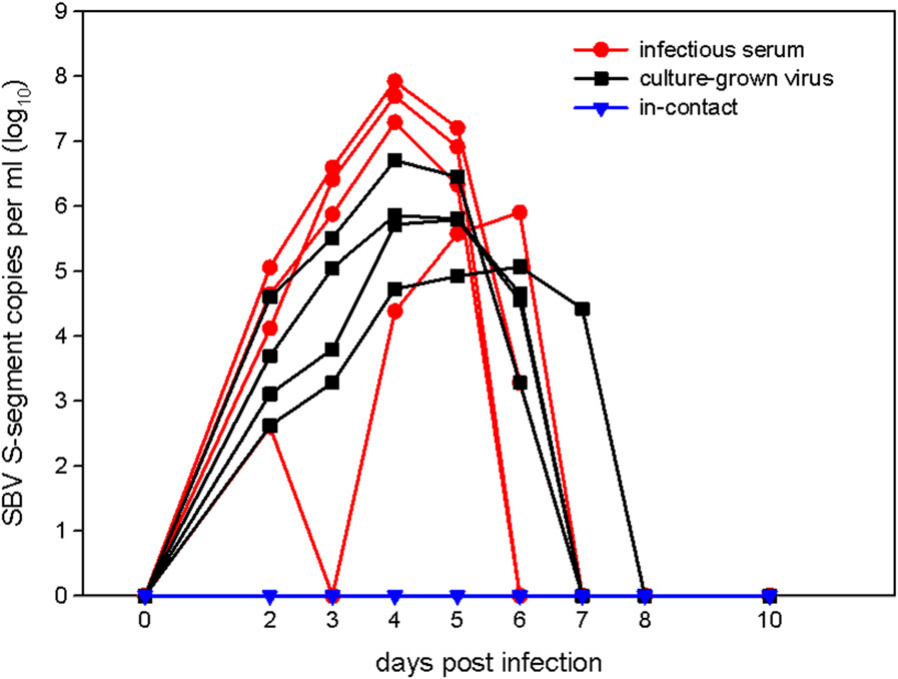
**Real-time RT-PCR results of cattle inoculated with infectious serum or cell culture supernatant.** All cattle, either inoculated with infectious serum or culture-grown virus, scored positive in the RT-qPCR for several days. In the in-contact animals SBV RNA was not detected at any time.

In the samples taken 2 days after infection, all cattle, either inoculated with infectious serum (group 1) or culture-grown virus (group 2), scored positive in the RT-qPCR for the first time. SBV genome remained detectable until day 5 in two out of four animals in group 1, until day 6 in the remaining two calves in group 1 and the three animals in group 2, whereas in the last calf in group 2 SBV genome was detected until day 7 (Figure 1). At peak RNAemia (4 dpi), there was no significant difference between the SBV RNA loads in groups 1 and 2 (*p* = 0.14, power = 0.519).

SBV RNA was not detected in the control animals at any time. Fever was not observed in any animal, but C08 developed mild diarrhea for two days.

Necropsy of the animals did not reveal any significant gross pathomorphological lesions. The mesenteric lymph nodes of C03 and C06-C08 were positive in the RT-qPCR (average Cq value: 34.8 ± 5.2). In addition, SBV RNA was found in the mandibular lymph nodes of C04 and C06 (Cq values: 39.0, 37.8), and the spleens of C05 and C06 (Cq values: 44.5, 36.9).

All cattle in group 1 and 2 were positive in the ELISA two weeks after infection, while all in-contact animals remained negative until the end of the study (data not shown).

### In vivo titration of the infectious serum

**Figure 2:**
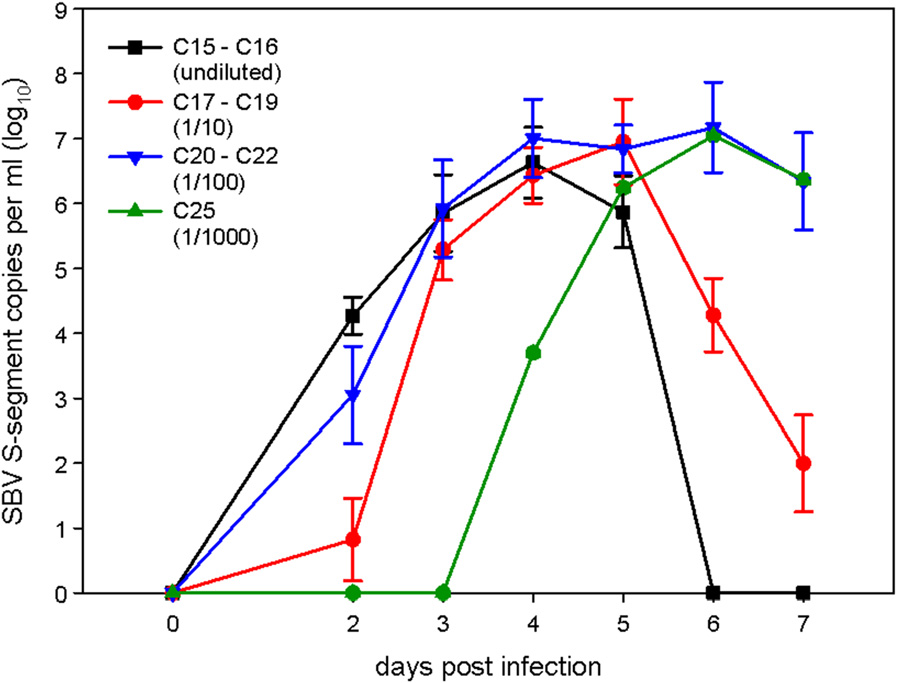
**Real-time RT-PCR results of cattle in the in-vivo titration experiment.** All animals inoculated with the undiluted serum, dilution 1/10 and 1/100 were positive for several days. Only one (C25) out of three animals inoculated with dilution 1/1000 scored positive.

In the in vivo titration experiment, both animals inoculated with the undiluted serum (C15/C16), as well as C17 (group B, 1/10), scored positive in the RT-qPCR between day 2 and 5. In C18 and C19 SBV genome was detectable for the first time on day 3. Animals in group C (1/100) scored positive on days 2 to 6, 4 to 7 and 3 to 7, respectively. In the group inoculated with dilution 1/1000 only one (C25) out of three animals was positive (Figure 2). Hence, the undiluted serum contained at least 10^2.83^ cattle infectious doses per mL (calculated by Spearman-Karber method). All cattle with positive RT-qPCR results (groups A, B, C and C25) seroconverted in the ELISA (data not shown). The mesenteric lymph nodes of these animals were positive in the RT-qPCR as well (average Cq value: 32.1 ± 2.4). Additionally, SBV RNA was detected in the mandibular lymph nodes of C16 C20, C22 and C25 (36.3 ± 3.3), the spleens of C17, C20 and C22 (39.6, 37.7, 44.8) and the tonsil of C19 (38.1).

## Discussion

The reliable induction of viremia is an important feature of challenge models for vector-borne diseases [9]. When viruses are passaged in cell culture, however, the positive selection of spontaneous mutations that promote replication in this environment can lead to changes in virus phenotype, including a reduced replication efficiency and virulence in the natural hosts. This has been demonstrated for several bunyaviruses [10,11] and is commonly exploited for the production of AKAV vaccines [12]. The genome stability of vector-borne viruses grown in culture can be promoted by switching between cell lines of mammalian and insect origin (mimicking natural host alternation) [13], but pertinent data for bunyaviruses are not available. In the present study, the replication of culture-grown SBV in cattle was reduced compared to a strain that had only been passaged in the natural host. While not statistically significant within the constraints of this experiment (small group size, high variability within groups), this difference provides a clear indication of the pitfalls of culture-based production of challenge inocula. The high level of infectivity in the tested serum batch offsets one of the major advantages of culture-grown viruses, namely the easier availability of large amounts of material. Based on our findings, cattle-derived infectious serum is a viable and robust option for a standardized SBV infection model, which is required for vaccine evaluation and pathogenesis studies.

## Competing interests

The authors declare that they have no competing interests.

## Authors’ contributions

Conceived and designed the experiments: KW ME BH MB. Performed the experiments: KW ME AB BH. Analyzed the data: KW ME AB. All authors read and approved the final manuscript.
